# On-wafer fabrication of cavity mirrors for InGaN-based laser diode grown on Si

**DOI:** 10.1038/s41598-018-26305-8

**Published:** 2018-05-21

**Authors:** Junlei He, Meixin Feng, Yaozong Zhong, Jin Wang, Rui Zhou, Hongwei Gao, Yu Zhou, Qian Sun, Jianxun Liu, Yingnan Huang, Shuming Zhang, Huaibing Wang, Masao Ikeda, Hui Yang

**Affiliations:** 1 Key Laboratory of Nanodevices and Applications, Suzhou Institute of Nano-Tech and Nano-Bionics, Chinese Academy of Sciences, Suzhou, 215123 China; 2 School of Nano Technology and Nano Bionics, University of Science and Technology of China, Hefei, 230026 China; 3 Suzhou Institute of Nano-Tech and Nano-Bionics, Chinese Academy of Sciences, Nanchang, 330200 China; 4 University of Science and Technology Beijing, Beijing, 100083 China

## Abstract

Direct bandgap III-V semiconductor lasers grown on silicon (Si) are highly desired for monolithic integration with Si photonics. Fabrication of semiconductor lasers with a Fabry–Pérot cavity usually includes facet cleavage, however, that is not compatible with on-chip photonic integration. Etching as an alternative approach holds a great advantage in preparing cavity mirrors with no need of breaking wafer into bars. However, gallium nitride (GaN) sidewalls prepared by dry etching often have a large roughness and etching damages, which would cause mirror loss due to optical scattering and carrier injection loss because of surface non-radiative recombination. A wet chemical polishing process of GaN sidewall facets formed by dry etching was studied in detail to remove the etching damages and smooth the vertical sidewalls. The wet chemical polishing technique combined with dry etching was successfully applied to the on-wafer fabrication of cavity mirrors, which enabled the realization of room temperature electrically injected InGaN-based laser diodes grown on Si.

## Introduction

Silicon photonics call for electrically injected semiconductor laser diodes (LDs) as on-chip light sources^1–3^. When grown on Si, III-nitride (Al, Ga, In)N semiconductors with a direct-band emission wavelength ranging from 0.2 to 1.8 μm offer a new approach for achieving on-chip lasers^4–7^. Facet cleavage as the conventional way of forming Fabry–Pérot cavity for semiconductor lasers, however, is not compatible with monolithic integration^8^. Therefore, it is indispensible to find a different way to prepare cavity mirrors for on-chip photonic integration with III-nitride waveguide to avoid the strong absorption of silicon^9–11^. Due to its chemical inertness of Ga-face GaN, dry etching is usually implemented in patterning InGaN-based LDs cavity mirrors^8,12–17^. However, GaN cavity mirrors fabricated by dry etching often suffer from surface roughness, poor steepness, and defects because of ion bombardment and mask erosion during dry etching^15,16^. These imperfections would result in optical loss due to light scattering and carrier injection loss because of surface non-radiative recombination, and hence, affect device performance and reliability of LDs^18^. To tackle the problems, it is necessary to develop an effective wet chemical technique to polish the etched facets^19,20^.

Potassium hydroxide (KOH) solution has been applied to the wet chemical etching of GaN-based LD cavity mirrors after dry etching^15–17^. However, KOH is a strong alkali and can cause triangular etching pits on the *m*-plane GaN surface even at a low temperature^21,22^. This may increase device leakage current and optical loss, as *m*-plane usually serves as the cavity mirrors for *c*-plane InGaN-based LDs. In addition, KOH solution attacks silicon dioxide (SiO_2_), which is commonly used as passivation layer for InGaN-based LDs. Compared to KOH solution, tetramethyl ammonium hydroxide (TMAH) is a kind of organic alkali, and has a more chemically stable etching process and a weaker chemical reaction with SiO_2_^22,23^. Hence, TMAH is a promising candidate for polishing the etched facets of InGaN-based LDs.

In this work, we studied in detail the morphology evolution of GaN *m*- and *a*-plane sidewalls during TMAH wet chemical etching. In addition, a simple atomic model was proposed to explain the wet chemical etching behavior of GaN *m-*plane sidewalls. The slanted rough *m*-plane sidewalls formed by inductively coupled plasma (ICP) dry etching can be smoothed out by the TMAH wet chemical polishing and work as cavity mirrors for InGaN-based LDs. And the threshold currents of the as-fabricated LDs were greatly reduced. The result is the realization of room temperature electrically injected InGaN-based LDs grown on Si, which may act as on-chip light sources for photonics integration.

## Results and Discussion

### The morphology evolution of GaN *m-* and *a-*plane sidewalls during TMAH wet chemical polishing

**Figure 1:**
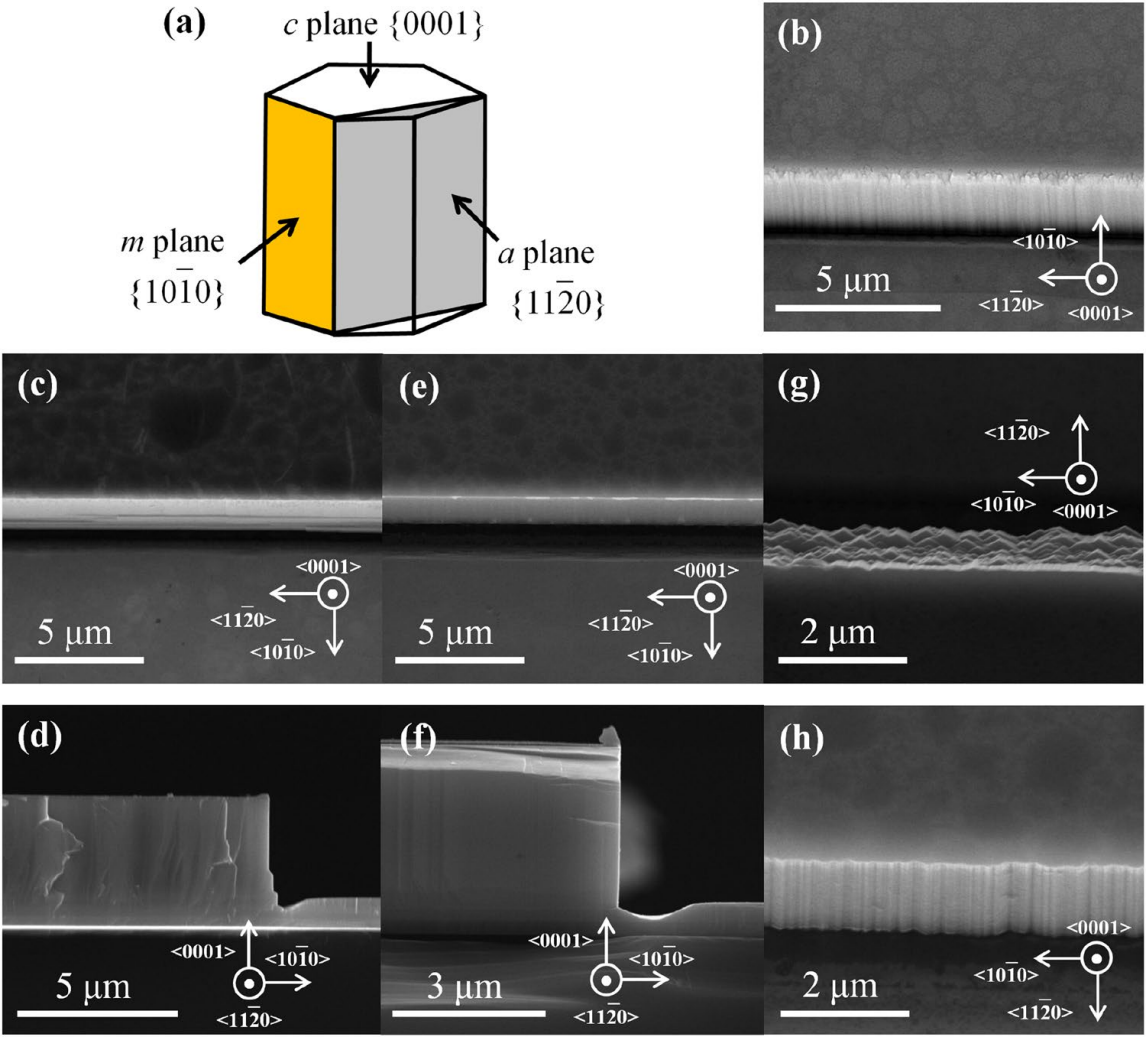
SEM images of GaN *m*- and *a*-plane sidewalls after being chemically polished in TMAH solution for various duration. (**a**) Schematic unit cell of hexagonal wurtzite structure. (**b**) The bird’s-eye view (tilted by 20°) SEM image of *m-*plane sidewall (the middle part) after the ICP dry etching. (**c**,**d**) The bird’s-eye view (tilted by 20°) and cross sectional images of *m*-plane sidewalls after a TMAH wet chemical polishing for 60 min. (**e**,**f**) The bird’s-eye view (tilted by 20°) and cross sectional images of *m*-plane sidewalls after a TMAH wet chemical polishing for 150 min. (**g**,**h**) The bird’s-eye view (tilted by 20°) SEM images of *a*-plane sidewalls after a TMAH wet chemical polishing for 60 and 150 min, respectively.

Before using the etching method to fabricate the cavity mirrors for InGaN-based LDs grown on Si, we study the morphology evolution of GaN *m-* and *a-*plane sidewalls during TMAH wet chemical polishing using 3-μm-thick crack-free high-quality GaN film. Several *c*-plane GaN samples grown on Si with stripe patterns along two different orientations, $< 11\overline{2}0 >$ and $< 10\overline{1}0 >$, covered by Ni mask and defined by photolithography, were first ICP etched, and then wet chemically polished in TMAH solution. Figure 1 shows a series of scanning electron microscope (SEM) images of these samples before and after the TMAH wet chemical polishing.

The bird’s-eye view SEM images of GaN *m*-plane sidewall after the ICP dry etching were taken at a tilted angle of 20°, so that the *m*-plane sidewall morphology could also be shown (Fig. 1b). The as-etched *m-*plane sidewall was rough with striations and slanted, which was typical after ICP dry etching. And the morphology of the as-etched *a-*plane GaN sidewall (not shown here) was nearly the same as that of *m*-plane. Figure 1c,d show the bird’s-eye view (tilted by 20°) and cross sectional SEM images of *m-*plane sidewalls after a TMAH wet polishing for 60 min. The *m*-plane sidewall became a lot smoother after the TMAH wet chemical polishing, only with some small cuboids at the bottom of the sidewall, as shown in Fig. 1c. It was not vertical toward the bottom of the sidewall, which is also clearly revealed in the cross sectional view (Fig. 1d). But as the TMAH wet polishing time was prolonged to 150 min, the entire *m*-plane sidewall surface became smooth and vertical (Fig. 1e,f).

The surface morphology evolution of GaN *a*-plane sidewalls during TMAH wet chemical polishing was distinctly different from that of *m*-plane sidewalls. After a TMAH wet polishing for 60 min, the *a-*plane sidewall was still rough with many triangular prism-like shapes, as shown in Fig. 1g. The top angle of these triangular prism-like shapes was ~120°. This is a typical morphology of GaN *a*-plane sidewall after being etched by alkaline solution, as also reported by many other groups^22,24–28^. With a prolonged wet polishing time of 150 min, those triangular prism-like shapes almost disappeared, but the *a*-plane sidewall surface was still quite rough (Fig. 1h), though the *a*-plane sidewall became vertical according to the cross sectional view (not shown here).

### A simplified atomic model of GaN *m-*plane sidewall during alkaline wet etching

In order to understand the surface morphology evolution of GaN *m*- and *a*-plane sidewalls during the TMAH wet chemical polishing, we examined the atomic structure of GaN *m*-plane sidewall in the presence of alkaline solution. It is well known that GaN can react with hydroxide ions (OH^−^) and produce GaO_x_, which can be dissolved in alkaline solution^19,20,29^. However, Ga-polar surface (+*c* plane) is much more chemically stable than N-polar surface (−*c* plane) in alkaline etching solution. This is because Ga-polar surface will be terminated with N atoms after the surface Ga atoms are removed, and each N atom on the surface has three negatively charged dangling bonds that can repel OH^−^ from further attacking the Ga-N bonds below^29^.

**Figure 2:**
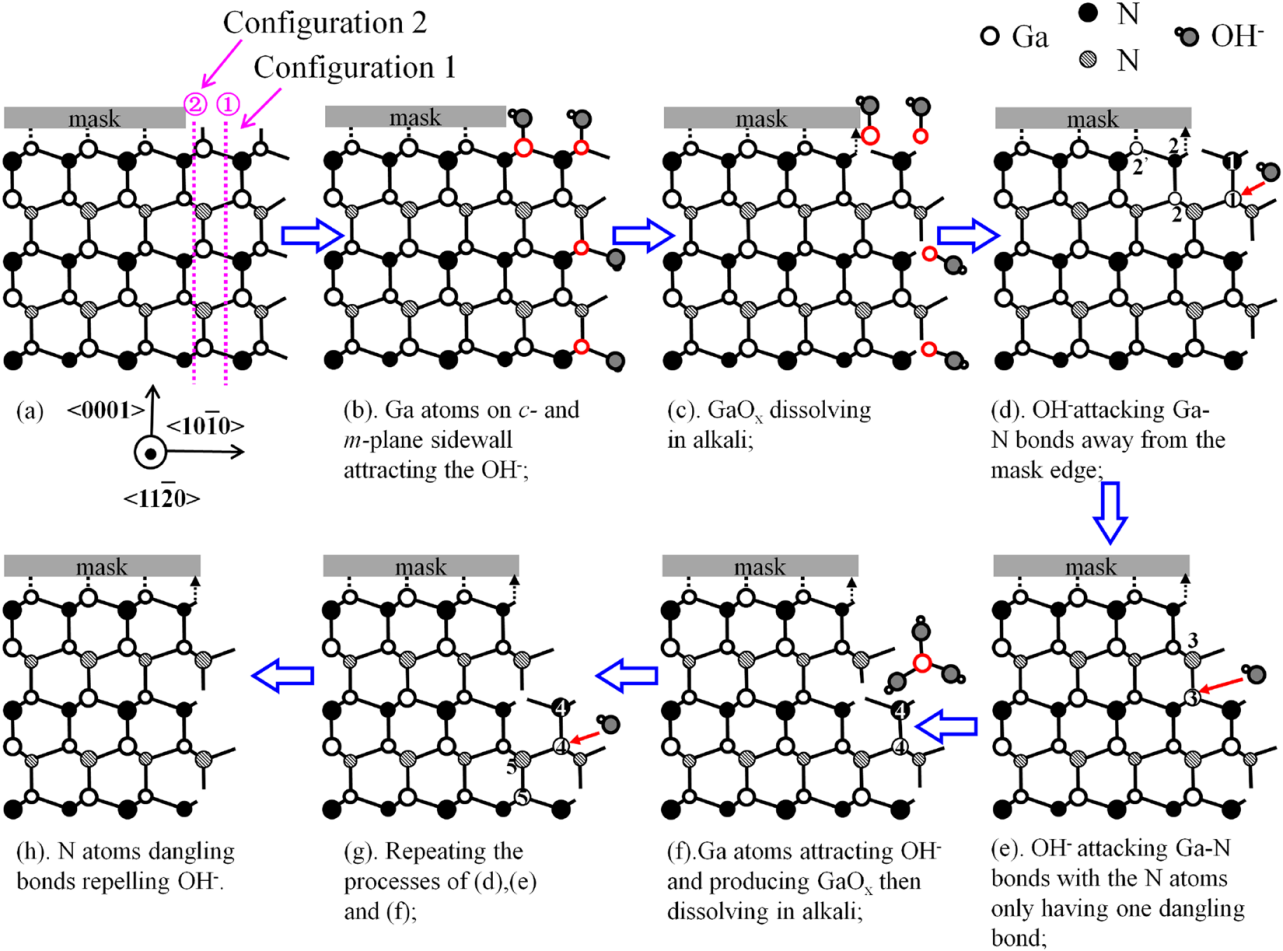
A simplified atomic model of TMAH wet chemical etching process of GaN *m*-plane sidewall under the mask, indicating the etching mechanism. The short dark line represents two bonds projected onto the paper plane, while the long dark line represents single bond parallel to the paper plane. The large symbols represent the atoms close to the paper plane, while the small ones represent the atoms far from the paper plane.

On GaN *m*-plane sidewall surface, there are two different kinds of surface configurations based on the number of surface dangling bonds, as shown in Fig. 2a. For configuration 1, each N atom represented by hollow circles filled with black oblique lines has only one dangling bond. For configuration 2, each N atom represented by solid black circles has two dangling bonds. A physical quantity called *etching barrier index* (EBI), which is the product of the planar atom density and the surface dangling bonds, was proposed by Yung *et al*. in ref.^30^ to describe the etching resistivity of each GaN plane in alkaline etching solution. The higher the EBI of the plane is, the more difficult the etching of the plane would be. The EBI for configuration 2 is twice higher than that of configuration 1, because they have the same planar atom density but a different number of negatively charged dangling bonds of N atoms. Therefore, it is more difficult for configuration 2 to be etched than configuration 1 in alkaline etching solution.

Figure 2 shows a simplified atomic structure of GaN *m*-plane sidewall during TMAH wet chemical etching process. First, the Ga atoms on the *c*-plane surface not covered by the mask and those on the *m*-plane sidewall having positively charged dangling bonds (Fig. 2a), can attract and react with OH^−^ in the TMAH alkaline solution (Fig. 2b), forming GaO_x_. Then GaO_x_ dissolves in the TMAH alkaline solution (Fig. 2c).

Secondly, the Ga atoms in position 1 on the *m*-plane sidewall surface are exposed to the solution and can be attacked by OH^−^ from the sidewall surface (Fig. 2d). And the N atoms above and below the Ga atoms in position 1 will be removed as well. The N atoms in position 2 right below the mask edge, as labeled in Fig. 2d, having two negatively charged dangling bonds, may form a kind of bond (indicated by the small arrow) with the mask. Those N atoms being anchored to the mask edge form a strong repelling force to OH^−^ from attacking the Ga atoms in position 2 and 2′. Therefore, the wet etching stops at the mask edge.

As shown in Fig. 2e, the Ga and N atoms in position 3 are in configuration 1 with a small EBI, and hence the Ga atoms at position 3 can be attacked by OH^−^ and become GaO_x_ dissolving in the alkaline etching solution (Fig. 2f). As shown in Fig. 2g, the Ga and the N atoms in position 4 will react with OH^−^ too, as they are equivalent to those at position 1. And the Ga and the N atoms in position 5 are equivalent to those at position 3, and hence will repeat the processes of Fig. 2e,f.

Finally, the GaN *m*-plane sidewall surface becomes the situation shown in Fig. 2h, where the N atoms both in configurations 1 and 2 around to protect the Ga atoms in configuration 2 from OH^−^ attacking. It means that the whole *m-*plane sidewall surface ends up with N atoms having negatively charged dangling bonds. Therefore, the *m*-plane sidewall becomes smooth and vertical (Fig. 2h).

In the case of GaN *a*-plane sidewall, on the other hand, the TMAH wet chemical etching would convert the sidewall surface into adjacent *m-*plane surfaces (Fig. 1a), forming triangular prism-like shapes with an angle of ~120° (Fig. 1g), and eventually many tiny zigzagged yet vertical *m-*plane sidewalls (Fig. 1h). Therefore, the wet etching mechanism of GaN *a*-plane sidewall follows that of *m-*plane sidewall.

### The cavity mirrors fabricated by etching method for InGaN-based LDs grown on Si

**Figure 3:**
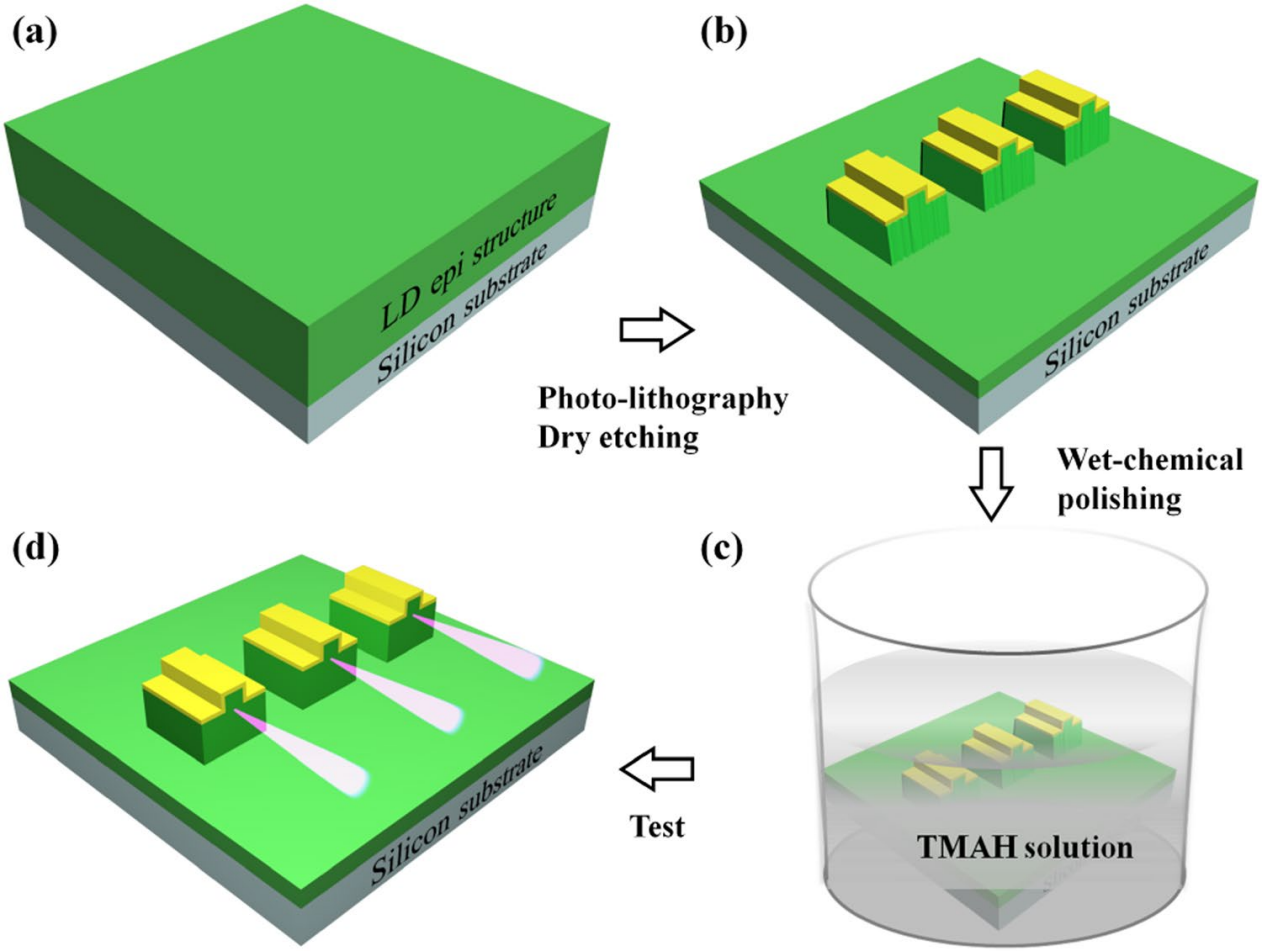
Simplified schematic diagram of the dry etching and the wet chemical polishing procedure applied to the fabrication of cavity mirrors of InGaN-based LDs grown on Si. (**a**) LD epitaxial structure was grown on Si substrate by MOCVD. (**b**) InGaN-based LD structure with the cavity mirrors fabricated by ICP dry etching (the n-type metal not shown in the diagram for simplicity). (**c**) The as-prepared cavity mirrors were chemically polished by TMAH solution. (**d**) Characterization of InGaN-based LDs grown on Si with the cavity mirrors polished.

Based on the understanding of GaN *m*-plane sidewall morphology evolution during the TMAH wet chemical etching, we fabricated the cavity mirrors of InGaN-based LDs grown on Si using the dry etching method together with the wet chemical polishing technique. The detailed epitaxial structure of InGaN-based LDs grown on Si can be found in ref.^4^. Figure 3a–d show the simplified schematic diagram of the dry etching and the wet chemical polishing procedure applied to the fabrication of cavity mirrors of InGaN-based LDs grown on Si. The fabrication process was described in detail in the method part. To be brief, the LD epitaxial structure was grown on Si substrate by MOCVD (Fig. 3a) and then the cavity mirrors were first fabricated by ICP dry etching (Fig. 3b) followed by soaking in the 25% TMAH solution at 85 °C for 150 min (Fig. 3c). Finally, the individual LD was tested on wafer (Fig. 3d).

**Figure 4:**
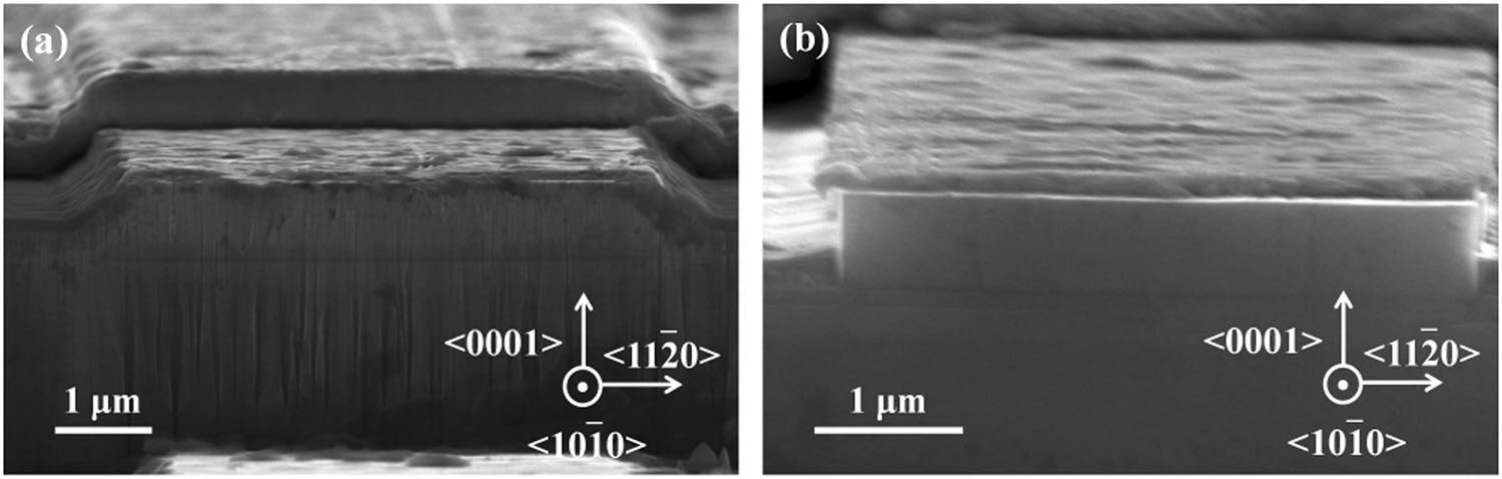
SEM images of the InGaN-based LDs grown on Si with cavity mirrors fabricated by the dry etching and wet chemical polishing technique. (**a**) ICP dry etching only, and (**b**) followed by a TMAH wet chemical polishing. The dark lines in Fig. 4b on the facet were due to contamination after the facet was exposed to the ambient.

Right after the ICP dry etching, the cavity mirrors were quite rough (Fig. 4a) as it observed by SEM. However, after the TMAH wet chemical polishing, the cavity mirrors became much smoother and vertical (Fig. 4b), which is very important for reducing the threshold current of LDs.

**Figure 5:**
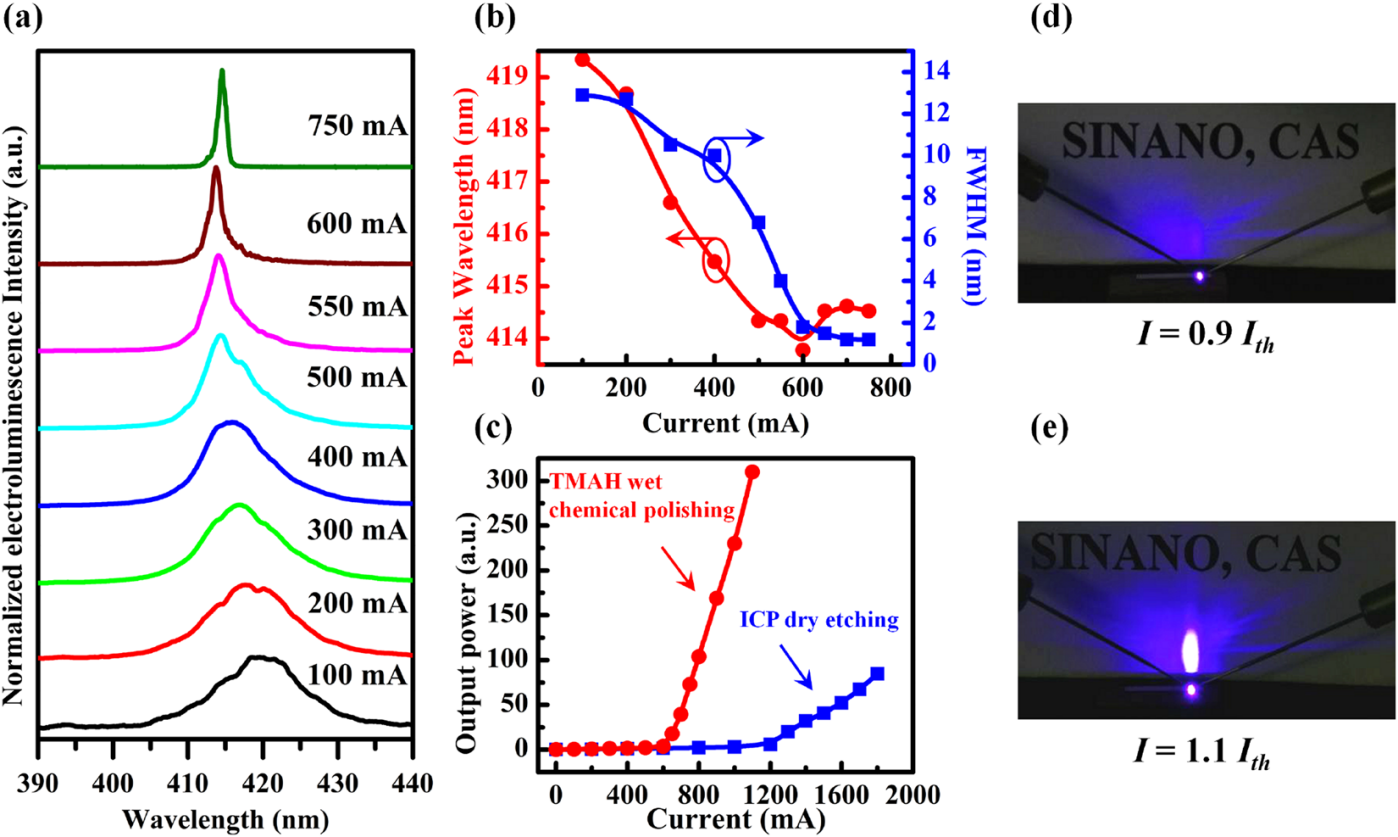
Room temperature device characteristics for one of InGaN-based LD grown on Si with the cavity mirrors fabricated by dry etching and wet chemical polish technique. (**a**) EL spectra measured under various pulsed electrical currents (a pulse width of 400 ns and a repetition rate of 10 kHz). (**b**) Peak wavelength and FWHM of the EL spectra as a function of the injection current. (**c**) EL light output power as a function of the injection current. (**d**) and (**e**), Far-field patterns observed below and above the threshold current by setting a sheet of white paper in front of the emitting facet of the LD. No coating was applied to the cavity mirrors of the LD.

The device characteristics for one of as-fabricated InGaN-based LDs grown on Si with the cavity mirrors prepared by the dry etching and the wet chemical polishing technique are shown in Fig. 5. Figure 5a presents the electroluminescence (EL) spectra of the LD under various pulsed injection currents at room temperature. As the injection current was gradually increased from 100 to 750 mA, the peak wavelength first blue-shifted from 419.3 to 413.8 nm due to the screening of quantum confined Stark effect by the injected carriers, and then red-shifted to 414.5 nm (Fig. 5b) because of the bandgap shrinkage caused by the increased junction temperature. Meanwhile, the full width at half maximum (FWHM) of the EL spectra quickly narrowed down to 1.8 nm at the injection current of 600 mA (Fig. 5b). In addition, the EL light output power increased quickly with the injection current at room temperature. The plot of the light output power as a function of the injection current exhibits a clear turning point at 600 mA for the LD with the cavity mirrors polished by the TMAH solution and 1200 mA for the LD with the cavity mirrors formed only by the ICP dry etching, as shown in Fig. 5c. Figure 5d,e are the far-field patterns of the edge emission from the device when the injection current was below and above the threshold, respectively. The above observations clearly indicate that an electrically injected lasing was achieved at room temperature for the InGaN-based LD grown on Si, and that the TMAH wet chemical polishing of the as-etched cavity mirrors can significantly reduce the threshold current.

**Figure 6:**
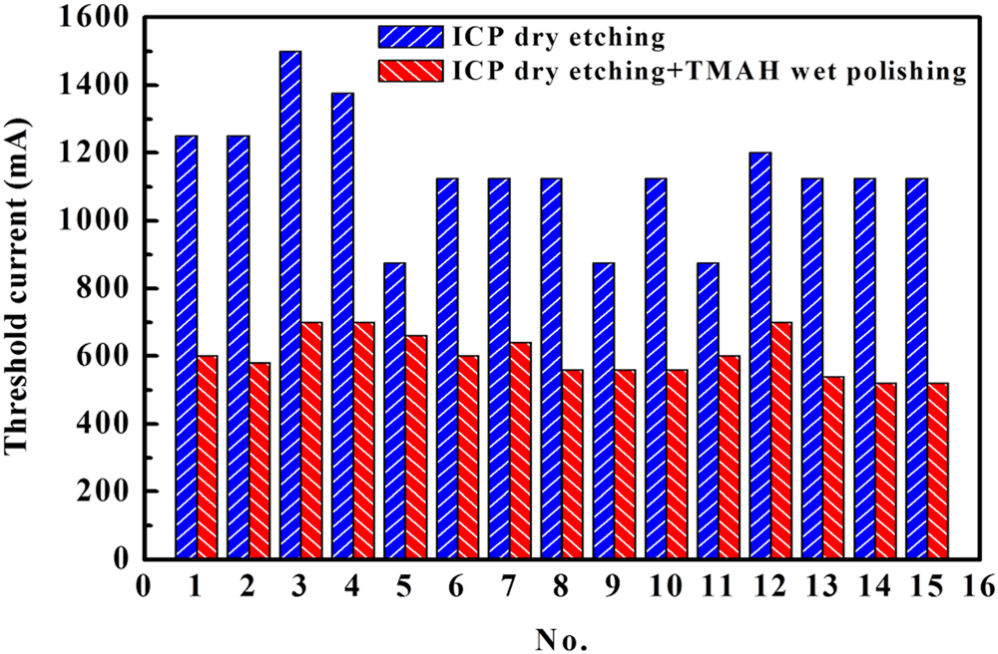
Statistical results of the lasing threshold currents for the as-fabricated InGaN-based LDs grown on Si with the cavity mirrors prepared by the ICP dry etching only (blue with left oblique lines) and polished by the TMAH solution (red with right oblique lines). No coating was applied to the cavity mirrors of the LDs.

In fact, we tested 15 InGaN-based LDs grown on Si under the same measurement condition to compare the threshold currents for the as-etched cavity mirrors before and after the TMAH wet chemical polishing. Figure 6 shows the statistical results of the lasing threshold currents for the 15 as-fabricated InGaN-based LDs with the cavity mirrors prepared by the ICP dry etching only and polished by the TMAH solution. The average threshold currents were 1138 and 600 mA before and after the TMAH wet chemical polishing, respectively, which displayed a 47% reduction in threshold current. The cavity mirrors fabricated by ICP dry etching only (Fig. 4a) were very rough and not vertical, and hence had a low reflectivity, which would lead to a scattering loss. Moreover, the ICP etching damages may act as non-radiative recombination centers, which is a loss channel for carrier injection, leading to an increase in threshold current. The TMAH wet chemical treatment can not only polish the as-etched *m*-plane cavity facets, making them smooth and vertical with a high reflectivity, but also effectively remove the ICP etching damages. Therefore, the threshold current of the as-produced LDs decreased greatly.

Different from the LDs in ref.^4^ whose cavity mirrors were fabricated by cleavage and deposited with highly reflective coatings to reduce mirror loss, the cavity mirrors of the InGaN-based LDs grown on Si in this study were formed by dry etching and polished by a wet chemical etching, with no reflective coatings applied. The mirror loss at the uncoated cavity mirrors of the LDs in this study resulted in a higher threshold current (about 600 mA, a peak current value under pulsed injection) than the LDs in ref.^4^. The lifetime of the as-fabricated laser was estimated to be 30 minutes under a pulsed injection prior to a significant decay in light output power.

The as-fabricated InGaN-based LD grown on Si in this study had a relatively high threshold current, which prevented a continuous-wave operation. And the relatively high threshold current was mainly due to the mirror loss with the uncoated cavity facets, imperfect active region, and high threading dislocation density (TDD) of ~6 × 10^8^ cm^−2^ (as compared with the homo-epitaxial devices)^4,10^. A study of GaN epitaxial lateral overgrowth on Si substrate, together with a further optimization of the active region and the doping of GaN-based LD, is underway to improve the performance and lifetime of InGaN-based LDs grown on Si for photonic integration.

In conclusion, we have developed an on-wafer fabrication technique for forming the cavity mirrors of InGaN-based lasers grown on Si. After a sufficient chemical polishing by TMAH wet solution, the as-etched GaN *m*-plane facets become smooth and vertical, and the ICP etching damages can be removed simultaneously. The result is the realization of room temperature electrically injected InGaN-based LDs grown on Si, which as on-chip light sources can be better monolithically integrated on Si than the conventional lasers prepared by facet cleavage.

## Methods

### TMAH wet chemical polishing process of GaN sidewalls

In the study of TMAH wet chemical etching process of GaN sidewalls, a 3-μm-thick crack-free high-quality GaN film (*c*-plane, (0001)) with an AlN nucleation layer and an Al-composition step-graded AlGaN buffer layer was grown on Si (111) substrates by metal-organic chemical vapor deposition (MOCVD)^31–33^. Stripe patterns were defined by a conventional photolithography procedure along two different orientations, $< 11\overline{2}0 >$ and $< 10\overline{1}0 >$. An optimized ICP dry etching condition with an ICP power of 300 W, an RF power of 100 W, and a flow rate of Cl_2_/BCl_3_ 10/25 sccm, was adopted to fabricate the stripe-like patterns on the GaN thin film. A 200-nm-thick nickel (Ni) was deposited as a hard mask for the ICP dry etching. After the ICP dry etching, the as-patterned GaN samples were soaked into a 25% TMAH solution at 85 °C to perform the wet chemical etching with a relatively high etching rate while avoiding severe water evaporation.

### The fabrication of cavity mirrors by etching method for InGaN-based LDs grown on Si

The detailed epitaxial structure of InGaN-based LDs grown on Si can be found in ref.^4^. Figure 3 shows a simplified schematic diagram of the dry etching and the wet chemical polishing procedure applied to the fabrication of cavity mirrors of the *c-*plane InGaN-based LDs grown on Si.

After the epitaxial growth of the LD structure on Si substrate (Fig. 3a), a metal stack of Pd/Pt/Au (30 nm/30 nm/50 nm) was firstly sputtered on top of the p-contact layer, followed by a thermal annealing at 600 °C for 90 seconds in a compressed air ambient. Then, photolithography and ion-beam etching were employed to form a 4 μm × 800 μm ridge structure. Subsequently, SiO_2_ was deposited on the surface of the wafer as an insulation layer with the ridge top window left open by a lift-off process. After that, a 200-nm-thick Ni was deposited as a hard mask for the ICP dry etching to form the cavity facets and expose the n-type GaN layer. Finally, Ti/Pt/Au (50 nm/100 nm/500 nm) metal stack was evaporated onto the ridge and the n-type GaN layer as p-type and n-type contact pads, respectively, as the device was fabricated in a co-planar structure with both p- and n-contact pads at the same side. Then, the Si substrate was thinned down to around 90 μm^4^. With ICP dry etching only, the as-fabricated cavity mirrors of the InGaN-based LDs grown on Si were rough and slanted (Fig. 3b). After being soaked in the 25% TMAH solution at 85 °C, the cavity mirrors were chemically polished (Fig. 3c).

### Characterization

The light output power was measured by putting a probe of an optical power meter (Thorlabs PM121D) about 5 mm away from the InGaN-based LDs. Only part of the light output was collected from the cavity mirrors under a pulsed injection current at room temperature. The pulse width was 400 ns, and the repetition rate was 10 kHz. The EL spectra of the InGaN-based LDs were measured by a fiber optic spectrometer (IdeaOptics FX4000) under a pulsed current injection. SEM (Quanta 400 FEG) was used to observe the GaN sidewall morphology and the cavity mirrors.

## References

[1] Soref, R. The past, present, and future of silicon photonics. *IEEE J. Sel. Top. Quantum Electron*. **1**2, 1678–1687 (2006).

[2] Chen R (2011). Nanolasers grown on silicon. Nat. Photonics.

[3] Chen SM (2016). Electrically pumped continuous-wave III–V quantum dot lasers on silicon. Nat. Photonics.

[4] Sun Y (2016). Room-temperature continuous-wave electrically injected InGaN-based laser directly grown on Si. Nat. Photonics.

[5] Feng M (2018). Room-temperature electrically pumped InGaN-based microdisk laser grown on Si. Optics express.

[6] Sun, Y. *et al*. Room-temperature continuous-wave electrically pumped InGaN/GaN quantum well blue laser diode directly grown on Si. *Light: Sci. Appl*. 10.1038/s41377-018-0008-y (2018). 10.1038/s41377-018-0008-y PMC6106987 30839586

[7] Feng M (2018). Room-temperature electrically injected AlGaN-based near ultraviolet laser grown on Si. ACS Photonics.

[8] Alex, B. *et al*. Etched facet technology for GaN and blue lasers. *Proc. of SPIE* **6121**, 61210P-1-8 (2006).

[9] Takuji S, Takashi S, Kazuhiro H (2015). Design, fabrication, and optical characteristics of freestanding GaN waveguides on silicon substrate. J. Vac. Sci. Technol. B.

[10] Feng, M. *et al*. On-chip integration of GaN-based laser, modulator, and photodetector grown on Si. *IEEE J. Sel. Top. Quantum Electron*. 10.1109/JSTQE.2018.2815906 (2018).

[11] Liu Q, Wang W (2018). Free-standing GaN grating couplers and rib waveguide for planar photonics at telecommunication wavelength. Opt. Laser Technol..

[12] Kneissl M (1998). Dry-etching and characterization of mirrors on III-nitride laser diodes from chemically assisted ion beam etching. J. Cryst. Growth.

[13] Scherer M (2001). Characterization of etched facets for GaN-based lasers. J. Cryst. Growth.

[14] Kao C-C (2004). Study of dry etching for GaN and InGaN-based laser structure using inductively coupled plasma reactive ion etching. Mater. Sci. Eng., B.

[15] Bottcher T (2002). Realization of a GaN Laser Diode with Wet Etched Facets. Phys. Status Solidi A.

[16] Miller MA (2009). Smooth and Vertical Facet Formation for AlGaN-Based Deep-UV Laser Diodes. J. Electron. Mater..

[17] Tian YD (2016). Stimulated emission at 272 nm from an AlxGa1−xN-based multiple-quantum-well laser with two-step etched facets. RSC Adv..

[18] Hsu PS (2013). Comparison of Polished and Dry Etched Semipolar (1122) III-Nitride Laser Facets. IEEE Photonics Technol. Lett..

[19] Stocker DA, Schubert EF, Redwing JM (1998). Crystallographic wet chemical etching of GaN. Appl. Phys. Lett..

[20] Zhuang D, Edgar JH (2005). Wet etching ofGaN, AlN, and SiC: a review. Mater. Sci. Eng. R, Rep..

[21] Jung Y (2016). Chemical etching behavior of non-polar GaN sidewalls. J. Cryst. Growth.

[22] M. SPR (2015). High-performance light-emitting diodes using hierarchical m-plane GaN nano-prism light extractors. J. Mater. Chem. C.

[23] Shikida M, Sato K, Tokoro K, Uchikawa D (2000). Differences in anisotropic etching properties of KOH and TMAH solutions. Sens. Actuators, A.

[24] Yue YZ (2014). Faceted sidewall etching of n-GaN on sapphire by photoelectrochemical wet processing. J. Vac. Sci. Technol., B.

[25] Itoh M (2006). Straight and Smooth Etching of GaN (1100) Plane by Combination of Reactive Ion Etching and KOH Wet Etching Techniques. Jpn. J. Appl. Phys..

[26] Baik KH (2011). Etched Surface Morphology of Heteroepitaxial Nonpolar (1120) and Semipolar (1122) GaN Films by Photoenhanced Chemical Wet Etching. J. Electrochem. Soc..

[27] Jung Y (2014). Chemical etching behaviors of semipolar (1122) and nonpolar (1120) gallium nitride films. Phys. Chem. Chem. Phys..

[28] Im KS (2016). Lateral GaN nanowire prepared by using two-step TMAH wet etching and HfO_2_ sidewall spacer. J. Cryst. Growth.

[29] Li DS (2001). Selective etching of GaN polar surface in potassium hydroxide solution studied by x-ray photoelectron spectroscopy. J. Appl. Phys..

[30] Lai YY (2016). The study of wet etching on GaN surface by potassium hydroxide solution. Res. Chem. Intermed..

[31] Leung B, Han J, Sun Q (2014). Strain relaxation and dislocation reduction in AlGaN step-graded buffer for crack-free GaN on Si (111). Phys. Status Solidi C.

[32] Li SM (2016). Off-state electrical breakdown of AlGaN/GaN/Ga(Al)N HEMT heterostructure grown on Si(111). AIP Adv..

[33] Zhong YZ (2017). Self-terminated etching of GaN with a high selectivity over AlGaN under inductively coupled Cl_2_/N_2_/O_2_ plasma with a low-energy ion bombardment. Appl. Surf. Sci..

